# Bacteriophage T5 gene D10 encodes a branch-migration protein

**DOI:** 10.1038/srep39414

**Published:** 2016-12-23

**Authors:** Io Nam Wong, Jon R. Sayers, Cyril M. Sanders

**Affiliations:** 1 Department of Oncology & Metabolism, University of Sheffield Medical School, Beech Hill Rd, Sheffield, S10 2RX, UK; 2 Department of Infection, Immunity & Cardiovascular Disease, University of Sheffield Medical School, Beech Hill Rd, Sheffield, S10 2RX, UK; 3 Sheffield Institute for Nucleic Acids, University of Sheffield Medical School, Beech Hill Rd, Sheffield, S10 2RX, UK

## Abstract

Helicases catalyze the unwinding of double-stranded nucleic acids where structure and phosphate backbone contacts, rather than nucleobase sequence, usually determines substrate specificity. We have expressed and purified a putative helicase encoded by the D10 gene of bacteriophage T5. Here we report that this hitherto uncharacterized protein possesses branch migration and DNA unwinding activity. The initiation of substrate unwinding showed some sequence dependency, while DNA binding and DNA-dependent ATP*ase* activity did not. DNA footprinting and purine-base interference assays demonstrated that D10 engages these substrates with a defined polarity that may be established by protein-nucleobase contacts. Bioinformatic analysis of the nucleotide databases revealed genes predicted to encode proteins related to D10 in archaebacteria, bacteriophages and in viruses known to infect a range of eukaryotic organisms.

Helicases play central roles in DNA replication ensuring fidelity^1^. Although a single replicative helicase may be responsible for unwinding the majority of a dsDNA genome, auxiliary helicases are necessary for replication re-start, DNA repair and recombination. Problems such as fork collapse or stalling at DNA lesions are likely to be routine occurrences during replication cycles, especially in organisms with large genomes. In *E. coli*, it has been estimated that up to 50% of initiation events from *ori*C may fail and lead to replication forks that must be re-activated independently of the normal initiation process^2^. The pathways that restore the replication fork and repair damage cope with a variety of DNA structures and often involve homologous genetic recombination. Accordingly, many helicases participating in DNA damage response pathways, including those with branch migration activity, are highly DNA structure-specific and it is this, rather than nucleotide sequence, that determines where and how they act on DNA^1^,^3^. Nevertheless, the unifying biochemical feature of all helicases is that energy from nucleotide hydrolysis is coupled to translocation and base-pair separation. Overall, this process is unbiased by nucleic acid sequence, consistent with structural and biochemical studies that show helicases interacting with nucleic acids principally via phosphodiester backbone contacts^4^,^5^,^6^.

DNA structure-specific helicases also regulate replication origins and this is best understood in bacteriophage systems. For example, bacteriophage T4 has two modes of replication initiation^7^,^8^,^9^,^10^. One is origin-dependent and depends on the 5′–3′, hexameric helicase T4 gp41 at the early stage of infection^11^,^12^. In order to accelerate the speed of replication and increase the burst size, a recombination-dependent replication (RDR) mode becomes predominant late in T4 infection. Here, products from the early origin-dependent DNA replication can invade each other by homologous recombination to generate D-loops for initiation of RDR^8^,^9^ and this requires the helicase activity of UvsW which promotes branch migration to stabilise the D-loop. UvsW also participates in the reactivation of stalled replication forks and branch migration of Holliday junctions^13^,^14^,^15^. Although best characterised in bacteriophage, RDR is not limited to prokaryotes and has been observed in mammalian viruses including HSV1 and papillomavirus^16^.

T5 is a lytic bacteriophage of *E. coli*^17^,^18^ that has a highly efficient replication cycle^19^,^20^,^21^,^22^. To date, only three proteins, D9 polymerase, D15 flap endonuclease and helicase D2 have been identified as replication enzymes in this bacteriophage^23^,^24^,^25^,^26^. Here we present biochemical analyses of a second bacteriophage T5 helicase, the D10 gene product^27^. The closest D10 protein homologs were identified as the *Archeoglobus* Xpb helicase, whose homologs have roles in transcription-coupled repair and nucleotide excision repair, and the UvsW protein encoded by bacteriophage T4. Additionally, homologs of the D10 protein were also found encoded within the genomes of several viruses capable of infecting single and multi-cellular eukaryotic organisms. We present biochemical studies showing that the D10 protein catalyses branch-migration *in vitro* and unwinds three- and four-strand DNA structures resembling DNA replication, recombination and repair intermediates. Intriguingly, unwinding of branched dsDNA structures by the D10 protein is highly sequence dependent and sensitive to purine modification interference, while DNA binding and DNA-dependent ATP*ase* activity are largely sequence independent. To our knowledge this is the first time that nucleobase sequence has been shown to influence unwinding by a DNA helicase and branch migration protein.

## Results

### Sequence analysis

**Figure 1:**
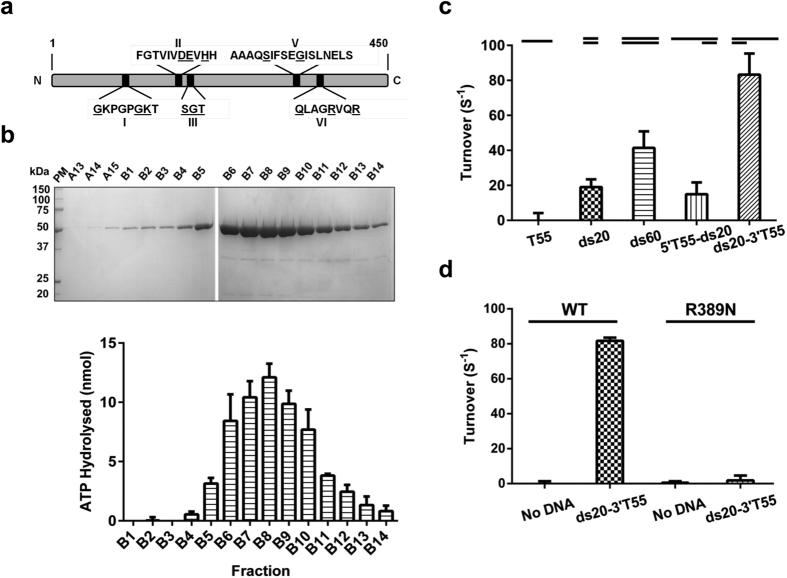
Conserved T5 D10 helicase motifs and ATP*ase* activity of purified protein. (**a**) Location of five conserved SF2 helicase motifs (I, II, III, V, VI) identified in D10. (**b**) Fractions containing D10 protein eluted from the final Superdex 75 SEC column analysed by SDS-PAGE (upper) and ATP*ase* activity of corresponding D10 peak fractions measured in the presence of DNA substrate ds20-3′T55 (20 bp dsDNA and a 3′ 55-base oligo-dT overhang, lower panel). (**c**) ATP*ase* activity of D10 (5 nM) determined in the presence of DNA substrates (5 nM): ssDNA, dsDNA and partial duplexes with a 20 bp and either a 5′ or 3′ 55 oligo-dT overhang (three repeats, mean and SD). (**d**) ATP*ase* activities of wild-type D10 and R389N (5 nM) determined in the absence or presence of 5 nM ds20-3′T55 (three repeats, mean and SD).

An analysis of the amino acid sequence of D10 revealed five conserved helicase motifs (Fig. 1a) belonging to the helicase superfamily 2 (SF2), including three universal helicase motifs, Walker A (motif I), Walker B (motif II) and an arginine finger (motif VI)^1^. Furthermore, we identified Xpb and UvsW helicases from *Archaeoglobus fulgidus* and bacteriophage T4 respectively, to be the closest characterised homologs of D10 (Supplementary Figure S1a). The D10 protein exhibits 20–30% identity (40–45% similarity) with the *A. fulgidus* Xpb and UvsW proteins including conserved helicase motifs and a basic/aromatic loop implicated in nucleic acid binding^28^. Intriguingly, aside from the expected orthologs and uncharacterised homologs present in T5-like bacteriophages, our analyses identified proteins encoded in the genomes of a number of viruses capable of infecting eukaryotic organisms as the closest homologs of the D10 protein. These included: Chorella and Marma viruses infecting the protozoans *Paramecium* and *Acanthamoeba* respectively; the Insectomime and *Armadillidium vulgare* iridescent viruses that infect insects; and the Singapore grouper iridovirus (Supplementary Figure S1b). A structural model of the D10 protein (Supplementary Figure S2) shows that the residues identified in the sequence alignments are present both in the core of the enzyme as well as the extended protein fold (Supplementary Figure S1b, and Video).

### Purification of T5 D10

The D10 helicase and a variant (R389N) were expressed in recombinant *E. coli* and purified by affinity, ion exchange and size exclusion chromatography (SEC), yielding approximately 0.01 and 0.06 mg of wild type D10 and D10 R389N protein respectively per gram of cells. Elution from the SEC column was consistent with a monomeric 50 kDa molecule and protein fractions contained a DNA-dependent ATP*ase* activity (Fig. 1b). This enzymatic activity corresponded exactly with the protein concentration of the fractions. Initial investigations with various linear DNA substrates including ssDNA (T55), blunt-ended dsDNA (20 and 60 bp), and partially single- and double-stranded molecules (ss-dsDNA) with either 5′ or 3′ ssDNA overhangs (20 bp and T55 ssDNA), demonstrated that only substrates with a duplex DNA component stimulated D10 ATP*ase* activity. Also, the substrate with 20 bp of dsDNA and a T55 3′ overhang (ds20-3′T55) was the most potent stimulator of ATP*ase* activity (Fig. 1c).[Fig f2][Fig f3] We also attempted to obtain D10 proteins with alterations in the three conserved motifs I, II and VI. However, despite successful cloning, only D10-R389N (motif VI) was successfully expressed and purified. ATP*ase* activity was almost completely abolished by the point mutation resulting in a substitution (R389N) in the conserved arginine finger motif (Fig. 1d), confirming that the DNA-dependent ATP*ase* activity is attributable to the D10 wild type protein.

### D10 Unwinds branched DNA substrates

**Figure 2 f2:**
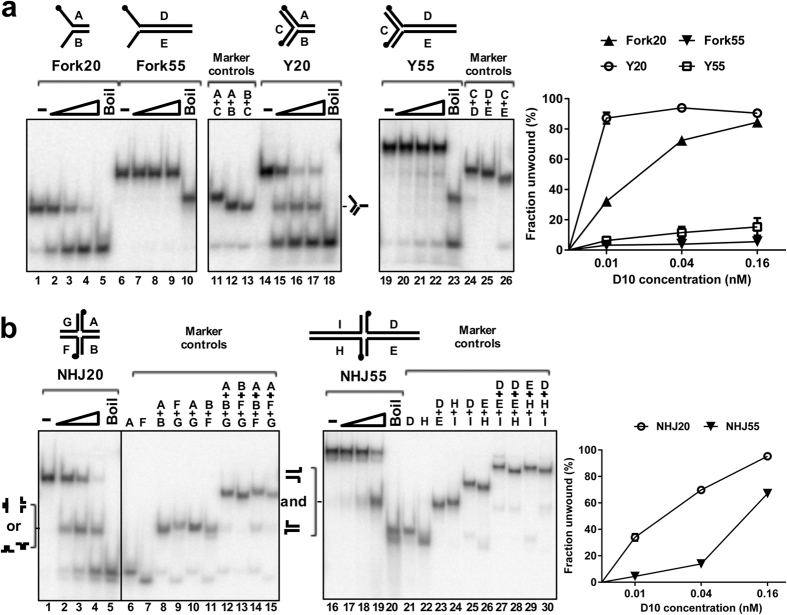
D10 unwinding of three- and four-way junction DNA. Substrates (0.1 nM) ^32^P end-labelled as indicated (black circles). (**a**) Unwinding of Y-shaped DNA molecules (0.01, 0.04 and 0.16 nM D10). Lanes 1, 6, 14 and 19, no protein/native substrates (–) and 5, 10, 18 and 23 show denatured controls (boil), as indicated in all subsequent Figures. Lanes 11–13 and 24–26 are markers of possible products. The graph on the right shows quantitative data for D10 unwinding (three repeats, mean and SD, standard deviation). (**b**) Unwinding of four-way (cruciform) DNA structures NHJ20 and NHJ55. Native and heat denatured substrates are indicated (lanes 1 and 16, 5 and 20) and lanes 6–15 and 21–30 are marker lanes for all possible products. Quantified data are shown in the graph on the right (3 repeats, mean and SD).

Even though the ATP*ase* activity of D10 was stimulated by ss-dsDNA substrates they were not unwound, nor could we detect a strand annealing activity for D10 that could confound interpretation of these results (Supplementary Figure S3 and Fig. 4a below). Since D10 shares sequence similarity with T4 UvsW, we considered whether D10 could, like UvsW^15^, unwind more complex branched DNA substrates with ss- and dsDNA arms (fork-like molecules/Y-junctions). D10 efficiently unwound a 20-bp duplex with two 20-base ssDNA arms (Fig. 2a, substrate Fork20), but not a similar molecule with the duplex arm extended to 55 bp by increasing the length of oligonucleotides A and B, even at the highest concentrations of protein tested (Fig. 2a, substrate Fork55). Similarly, a dsDNA Y-junction with three 20-bp duplex arms was unwound efficiently, but not the substrate with one dsDNA arm extended to 55 bp (Fig. 2a, substrates Y20, and Y55, respectively). Both of these substrates, Fork20 and Y20, were only unwound by D10 in the presence of ATP (Supplementary Figure S4). In addition, for Y20, D10 preferentially dissociated only one of the three strands (oligo A) from the substrate, as described in further detail below. Thus, unlike UvsW, D10 can unwind DNA Y-junctions, although the length of the dsDNA substrate appears to impose limits on the ability of D10 to unwind certain structures *in vitro*.

Next, we tested the ability of the D10 protein to unwind synthetic cruciform (four-way) non-homologous Holliday junction substrates, as these are unwound by T4 UvsW. Substrate NHJ20, with four 20 bp non-homologous dsDNA arms, was unwound generating two sets of products, forked DNA and ssDNA (Fig. 2b). However, unwinding of the substrate NHJ55 with two 55 bp and two 20 bp dsDNA arms generated only one kind of product, a forked DNA with only a 55 bp dsDNA arm (Fig. 2b). Furthermore, the D10 ATP*ase*-deficient variant R389N failed to unwind NHJ20 but retained equivalent DNA binding activity to wild type, confirming that the observed reaction products result from the enzymatic action of D10 (Supplementary Figure S5).

**Figure 3 f3:**
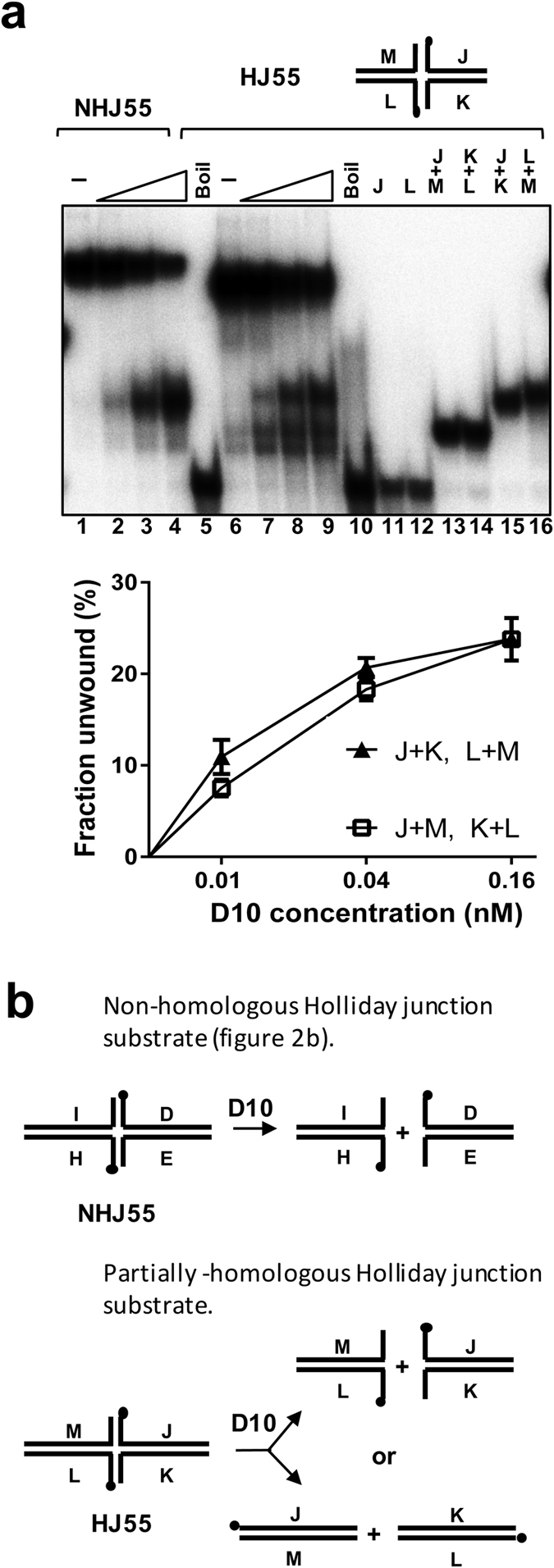
D10 Unwinding of a partially homologous Holliday junction substrate. (**a**) Substrate HJ55 (0.1 nM) was unwound by D10 (0.01, 0.04 and 0.16 nM, lanes 7–9) resulting in two kinds of products, fork DNA and recombined duplex DNA, identified from marker lanes 11–16. Lanes 1–5 show the unwinding and control reactions for the substrate NHJ55 run in parallel where only one class of product was observed as demonstrated in Figure 2. The graph below gives the percentage of each product class. (**b**) Schematic representation for D10 unwinding of non-homologous (NHJ55) and partially-homologous (HJ55) substrates.

As mentioned above, one feature of our observations was that dsDNA length appears to impose a restriction on the ability of D10 to unwind certain test substrates (Fig. 2a). One possibility is that the enzyme can only melt ≤20 bp of duplex DNA at, or close to, the substrate junction point. Alternatively, the longer DNA arms could re-anneal before completely dissociated or, envisioning a branch migration activity, the non-homologous nature of the test substrate could restrict the length of dsDNA that can be unwound. To investigate these possibilities, we constructed a close mimic of a natural Holliday junction substrate, consisting of a pair of long (55 bp) homologous duplex arms and a pair of short (20 bp) heterologous duplex arms (substrate HJ55, Fig. 3). In comparison to NHJ55, D10 converted HJ55 with two homologous 55-base-pair dsDNA arms into two different kinds of products; fork DNA and recombined linear duplex DNA (Fig. 3a), resulting from unwinding of HJ55 in either of two possible orientations. D10 therefore has branch-migration activity and can unwind DNA lengths greater than 55 base pairs.

### DNA Sequence context and unwinding activity of D10

**Figure 4:**
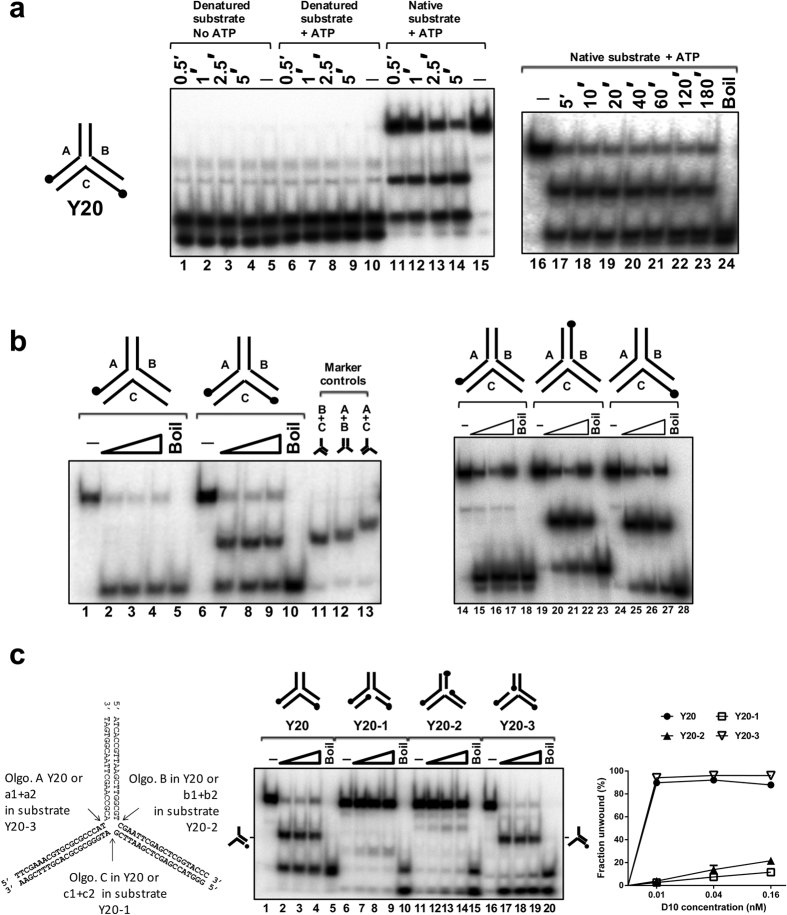
D10 processing of substrate Y20. **(a)** Time-course for unwinding of Y20 (0.1 nM) with oligonucleotides A and C ^32^P end-labeled. Analyzed in lanes 1–10 are reaction products where substrate Y20 was heat denatured before addition to the reactions. D10 failed to catalyze strand annealing with or without ATP present, as indicated. Lanes 11 to 15 show accumulation of unwinding products of native Y20 substrate in parallel reactions with ATP sampled in a 5 minute time frame (0.1 nM substrate, 0.04 nM D10. Lanes 5, 10 and 15 are no protein controls). Lanes 16 to 24 show product accumulation over an extended time frame up to 180 minutes. (**b**) Helicase reactions showing preferential displacement of oligonucleotide A from substrate Y20. Lanes 1–13, helicase reactions with Y20 (0.1 nM) radiolabelled on strand A only or strands A and C, demonstrating that oligonucleotide A is preferentially displaced. Lanes 1 and 6, no protein control (–); lanes 5 and 10, heat-denatured substrate control (Boil); lanes 2–4 and lanes 7–9, with D10 (0.01, 0.04 and 0.16 nM); lanes 11–13, markers for possible products. Lanes 14–28 helicase reactions performed with separately labeled oligonucleotides A, B and C under the same conditions. (**c**) Processing of Y20 substrates with a nick positioned in each of the oligonucleotide as illustrated on the left. Centre, helicase reactions (0.1 nM substrate, 0.01, 0.04, 0.16 nM D10) for substrates Y20, Y20-1, Y20-2 and Y20-3 with the labelled oligonucleotides indicated for each substrate (black circle). Statistical data (three repeats, mean and SD) are shown in the graph on the right.

The data described above identified three- and four-way branched DNA structures, as substrates for the D10 helicase and indicated that D10 has branch migration activity. However, these data also show that D10 unwinding action is influenced by additional parameters other than DNA structure and length. For example, D10 could completely dissociate the substrate Fork20 with a 20 bp duplex and two 20 base ssDNA arms (Fig. 2a). It could also displace a 40 base oligonucleotide from substrate Y20 with three 20 bp duplex DNA arms (Fig. 2a), but the co-product that resembles Fork20 (20 bp and two 20 base ssDNA arms) was only poorly unwound, even in an extended time course (Fig. 4a). Furthermore, the relative mobility of the unwinding products indicated that there was a very strong bias as to which 40 base oligonucleotide was displaced from the Y20 substrate (Fig. 2a, lanes 11–13 compared to 15–17), which we unambiguously identified as oligonucleotide A (Fig. 4b). There are two possibilities for interpreting these data: either DNA sequence or secondary structure formed by ssDNA arms can influence D10 unwinding activity.

In order to minimize undesirable secondary structure and further explore the potential sequence selectivity of DNA unwinding in the D10 enzyme, the substrate Y20 was compared to the same substrates but with a nick at one of the three junction branch points in each substrate (Fig. 4c). According to Sabir and co-workers, all these three nicked substrates should have the same fully complementary largely symmetrical conformation without coaxial stacking of arms^29^. Thus, we expected all the nicked Y-junctions to be unwound to similar extents. Surprisingly however, as shown in the electrophoretogram in Fig. 4c, compared to Y20, only Y20-3 (oligo A nicked) was unwound efficiently. As summarised graphically (Fig. 4c), practically all of substrates Y20 and Y20-3 were unwound at the lowest protein concentration tested, but the extents of unwinding of Y20-1 and Y20-2 were at least 20 fold lower. The reaction products of Y20 and Y20-3 were also both consistent with displacement of the sequences corresponding to oligonucleotide A; two oligonucleotides in the case of substrate Y20-3 (a1 and a2). However, although poorly unwound, the reaction products of Y20-1 and Y20-2 were not consistent with preferential displacement of this sequence (Fig. 4c and Supplementary Figure S6a).

To further explore the basis of this substrate-specific DNA unwinding we tested whether the various Y-junctions differed in their binding affinity for the D10 protein and whether they could stimulate DNA-dependent ATP*ase* activity. Substrates Y20, Y20-1 and Y20-2 showed little detectable difference in their binding affinities for D10, while Y20-3 (oligonucleotide A nicked) showed a modest decrease in affinity (~2 fold) as judged by the sensitivity of substrate binding to the addition of non-specific competitor DNA in the reaction (Supplementary Figure S6b). Furthermore, all Y-junctions with and without nicks stimulated the DNA-dependent ATP*ase* activity of D10 to similar extents (Supplementary Figure S6d). Thus, displacement of oligonucleotides A or a1 and a2 from Y20 is dependent on the strand continuity of oligonucleotides B and C, indicating a precise orientation of D10-substrate unwinding relative to DNA sequence.

### High-resolution footprinting of D10 bound to a dsDNA Y-junction

**Figure 5:**
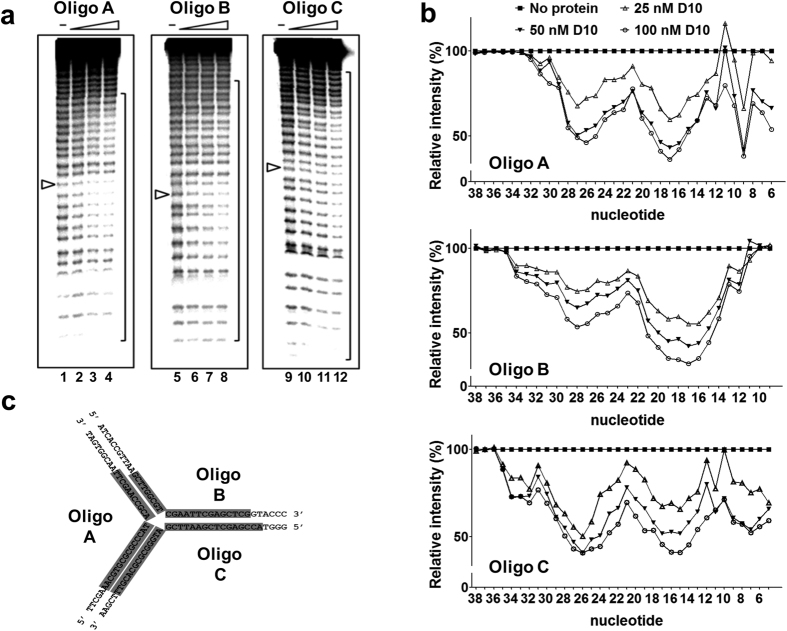
Hydroxyl radical footprinting of the D10-Y20 DNA complex. (**a**) Either oligonucleotide A, B or C of the Y20 substrate was ^32^P end-labelled. Binding reactions contained 10 nM substrate and 0, 25, 50, 100 nM D10. The 20^th^ nucleotide at the branch point of each strand are indicated by arrows. (**b**) Regions of protection were determined by comparing densitometric tracings with and without D10 at the indicated protein concentrations. For each nucleotide position, the intensity of each band was compared directly to the intensity of the reaction with no protein (100% intensity) to give the relative intensity of protection as a function of increasing protein concentration. (**c**) The regions of protection are schematically represented on the Y20 sequence.

Next, we performed high-resolution hydroxyl radical (OH•) footprinting to investigate the binding of D10 to substrate Y20. Three D10 binding reactions, each with a different strand of Y-20 labelled, were assembled and the OH• was generated by Fenton’s reaction. After limited cleavage, the DNA products were recovered, separated on a sequencing gel analysed by quantitative phosphorimaging. D10 protected the 23–30 bases, approximately centred around the junction point in each DNA strand of Y20 (Fig. 5a).

An analysis of the protection pattern with increasing protein concentration (Fig. 5b) suggested that protein binding to the Y-junction was progressive, without demonstrating a significant bias for any strand or arm of the substrate. However, although the Y-junction substrate has trilateral symmetry the protection pattern, summarised in Fig. 5c, did not display such symmetry, with the length of DNA protected on oligonucleotide C (30 nt.) more extensive than that of A (26 nt.) or B (23 nt.).

### DEPC Interference analysis of D10-Y20 DNA unwinding reactions

**Figure 6:**
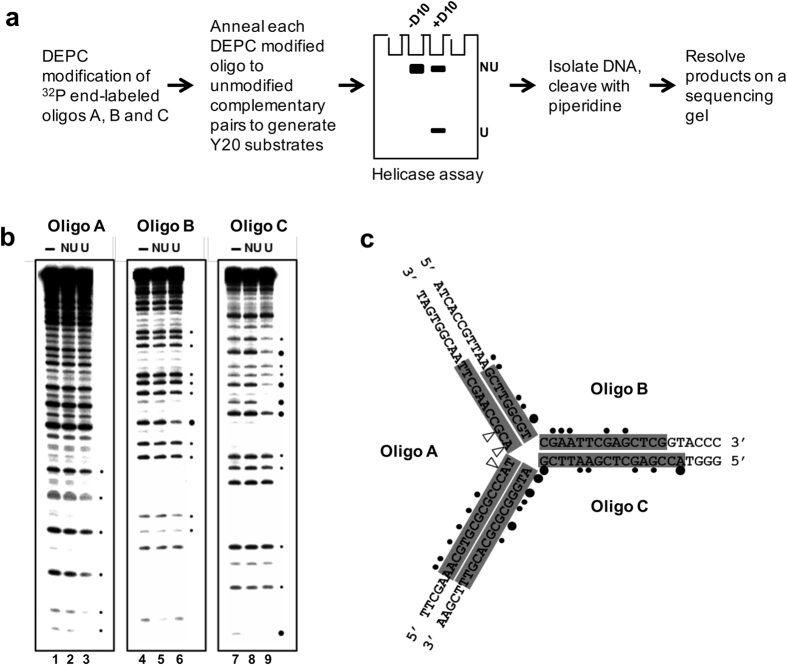
DEPC interference analysis of the unwinding of Y20 by D10. (**a**) Schematic diagram of the experimental procedure (NU = substrate not unwound and U = unwound substrate). (**b**) The helicase reactions were performed with D10 and one each of the three strands of substrates Y20 was ^32^P end-labelled and DEPC-modified. Positions of interference are indicated (circles). (**c**) The OH protection is shaded in grey and the positions of modification-interference within the Y20 substrate indicated (black dots, interference; small symbol, less than 40% change; large symbol, 40–70% change).

Diethyl pyrocarbonate (DEPC) carbethoxylates N7 of purines and the presence of this adduct can interfere with close protein-DNA base contacts required for substrate unwinding^30^. Y20 substrates were generated with one strand end-labelled with^32^ P and modified with DEPC and used in unwinding reactions. After electrophoresis, the intact substrate and product were recovered, cleaved with piperidine and analysed on a sequencing gel (Fig. 6a), as above. Carbethoxylation of several purine residues on each strand inhibited unwinding (Fig. 6b). Quantification of the data revealed that the number of purines and the magnitude of the resulting effects of DEPC modification on inhibition of unwinding are far greater for oligonucleotides B and C, compared to the displaced oligonucleotide A, where they are also all present exclusively on the 5′ half of oligonucleotide A. Furthermore, the three purines at or close to the junction point of oligonucleotide A (indicated with arrows in Fig. 6c) do not alter the efficiency of the unwinding reaction when carbethoxylated, while the purines in similar positions in oligonucleotide B and C show significant effects on unwinding when modified. These data imply that base (purine) contacts in oligonucleotide B and C, particularly those close to the junction point, influence the displacement of oligonucleotide A from the substrate. There is therefore a correlation with the data in Fig. 4, where nicking of oligonucleotides B or C at the junction point inhibits the displacement of oligonucleotide A.

### Initiation of unwinding of duplex Y Junctions is sequence dependent

**Figure 7:**
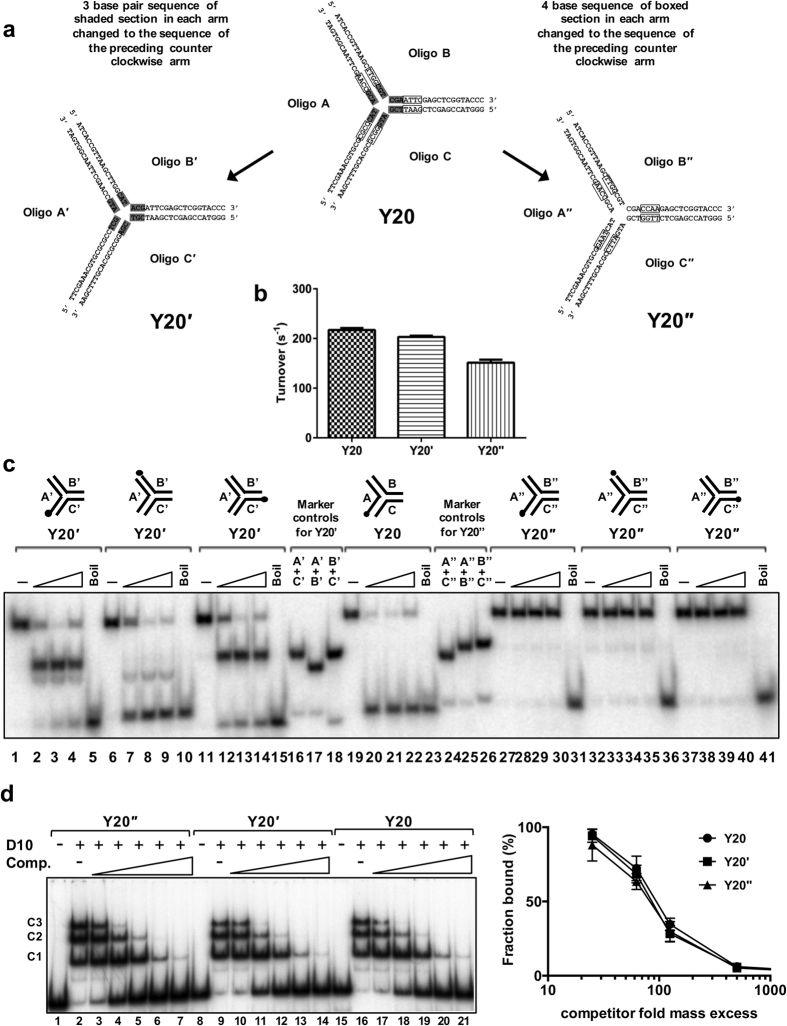
Unwinding and DNA binding of Y20 with sequence alterations. (**a**) The sequences for generating substrates Y20′ and Y20′′ were derived by rotating Section 1 (shaded; 3 bp; positions 1–3 from the junction point) and Section 2 (boxed; 4 bp; positions 4–7) of Y20 120° clockwise to adjacent arms. (**b**) ATP*ase* activity of D10 (5 nM) was determined in the presence of Y20, Y20′ or Y20′′ (5 nM). (**c)** D10 displaced all oligonucleotide from Y20′, but oligonucleotide B in preference (lanes 1–18; 16–18 are markers for the possible products), but was incapable of unwinding Y20′′ (lanes 24–41; lanes 24–26 are markers for the possible products). (**d**) Gel-shift assay (0.1 nM substrate, 0.4 nM D10) showing D10 binding to Y20′′, Y20′ and Y20 (lanes 2, 9 and 16 compared to lanes 1, 8 and 15, no protein control (–). Binding reactions were also performed in the presence of increasing amounts of poly d(AT) competitor (2–200 ng) for each substrate, lanes 3–7 (Y20′′), lanes 10–14 (Y20′), and lanes 17–20 (Y20) and the quantified data (n=4, mean and standard deviation) are shown in the graph.

The data above indicate that DNA base sequence is a major determinant in the efficiency of D10 Y-junction unwinding. Without *a priori* knowledge of D10 sequence requirements a corollary to this observation is that the initial choice of substrate sequence was fortuitous in terms of revealing its ability to be unwound. We therefore altered Y20 in two ways to test whether sequence alterations alter its susceptibility to D10-mediated unwinding. The alterations made were limited to the 7 base pairs of each fork arm closest to the junction point where DEPC interference was most apparent in helicase assays. To generate the oligonucleotide sequences for substrate Y20′, as illustrated in Fig. 7a, the sequence of the 3 base pairs at the junction point of each dsDNA arm of Y20 (‘section 1’) was changed to the sequence of the preceding, counter clockwise, arm *i.e.* a rotational transposition of the 3 bp sequence of each arm in the clockwise direction. To generate the sequences for substrate Y20′′, the nucleotide base pair sequence at positions 4–7 of each oligonucleotide (‘section 2’) were transposed similarly. Such sequence transformations had only a minimal impact on ATP*ase* activity with only Y20′′ showing a slightly reduced (~30%) ability to stimulate the rate of D10-catalysed ATP hydrolysis (Fig. 7b).

Next, each oligonucledotide of the substrates was labelled individually in order to determine how they are processed in helicase assays (Fig. 7c). For Y20′, lanes 1–18 and control reactions, the sequence alteration at the branch point shifted the preference for displacement predominantly from the context of oligo A to oligo B. In addition, the other oligonucleotides were displaced from the substrate but with reduced efficiency. Overall, Y20′ was unwound with similar efficiency to Y20 (lanes 19–23). In contrast, the sequence transformation in substrate Y20′′ resulted in complete inhibition of D10 catalysed unwinding. Thus, since near complete unwinding of substrate Y20 is achieved in less than 5 minutes (Fig. 4a) and no unwinding of substrate Y20′′ was observed in 20 minutes, small sequence alterations have very large effects on substrate unwinding. There is no correlation between oligonucleotide GC base content in the first 6 residues either side of the fork junction and strand displacement. For example, oligonucleotide A is efficiently and exclusively displaced from substrate Y20 and in oligonucleotide A four of the six nucleotides are G or C residues in each of the first six nucleotides either side of the junction point (eight in total) while in oligonucleotides B and C there are a total of six G or C residues, also considering the six nucleotides either side of the junction point. Thus, reduced thermal stability is unlikely to account for selective displacement of oligonucleotide A form Y20. Also, although there are two A:T base pairs at the junction formed by oligonucleotide A and only one for oligonucleotides B and C it is known that each of the first bases at the branch point in such three-way junctions are unpaired^29^ and therefore unlikely to make any contribution to junction stability and selective unwinding. This is confirmed by the complete failure of D10 to displace oligonucleotide A′′ from substrate Y′′, where the two A:T base pairs at the fork junction point are maintained.

Furthermore, in the absence of ATP/Mg^2+^, all three substrates (Y20, Y20′ and Y20′′) were bound by D10 to similar extents and three principal complexes (C1–3) were observed when all substrate was bound by protein, as shown in Fig. 7d lanes 2, 9 and 16. D10 binding to each Y-junction substrate demonstrated very similar sensitivity to the addition of increasing amounts of non-specific competitor DNA poly d(AT), indicating a near identical D10 binding affinity for all substrates.

## Discussion

Like its closest characterised homologue T4 UvsW, T5 D10 can unwind synthetic three, and four-way branched dsDNA substrates by branch migration. However, unlike UvsW and the other known branch migration enzymes including *E. coli* RecG^31^ and RuvAB^32^, helicase action is influenced by the sequence of the branched dsDNA substrates. Substrate sequence had a significant effect on the ability of D10 to unwind simple Y-shaped molecules with two ssDNA tails (Fig. 2) as well as how branched substrates with three and four dsDNA arms were processed. Unlike four-way dsDNA junctions, the arms of Y-shaped dsDNA molecules have been shown to behave much like duplex DNA and are relatively insensitive to Mg^2+^ induced structural perturbations due to base stacking^29^. This and the observation that a nick in any one DNA strand at the junction point, that would be expected to relax any conformational constraints induced by metal ions at the junction, does not alter selective strand displacement, reinforcing the notion that D10 is directly sensitive to substrate sequence. Moreover, the effects of breaking the phosphodiester backbone at the junction point are consistent with the notion that productive unwinding is dependent on sequence orientated D10-substrate interactions (see below). In contrast, DNA binding and DNA-dependent ATP*ase* activities were relatively independent of the substrate sequence or insensitive to nicking of the DNA backbone at the junction point. To our knowledge this is the first time that significant sequence-selective initiation of unwinding has been observed for any DNA helicase and branch migration protein.

Primarily, DNA structure rather than base sequence recognition determines where helicases initiate unwinding. Helicases involved in DNA repair such as BLM, WRN^3^,^33^ and PIF1^34^ are recruited to branched DNA structures that form during DNA repair. The hexameric replicative helicases are usually loaded on to single-stranded DNA (ssDNA) where their motor domains make DNA base-independent interactions withss DNA^6^,^35^. For *E. coli* DnaB helicase, the ssDNA structure is prepared at *OriC* by the initiator protein DnaA^36^. For the replicative helicases of SV40 and papillomavirus, large T-antigen and E1 respectively, separate modules for sequence specific origin (*ori*) recognition and helicase action function independently and the initiator binding sequence is separate from the site of helicase binding^37^. In contrast, the protein sequence alignment of D10 with both UvsW and XPB are not consistent with a truly modular organisation and the putative DNA binding site of UvsW, an arginine/aromatic rich (basic) loop conserved in D10 (Supplementary movie file), is best described as a DNA binding segment within the helicase fold. Furthermore, in D10-three-way dsDNA fork complexes the unwinding junction and sites of close DNA base-protein contact that determine substrate unwinding appear coincident (see below).

**Figure 8:**
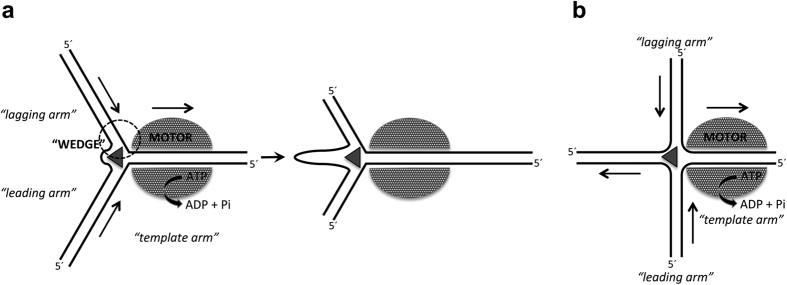
Model for D10 unwinding of branched dsDNA structures. (**a**) Unwinding of substrate Y20 resembles processing of a stalled replication fork to a “chicken foot” intermediate by RecG. A fork separation “wedge” acts at the junction to unwind nascent leading-, and lagging-strand DNA as the helicase motor moves the “template arm” through the complex. In D10, DNA binding segments of or in proximity to the wedge can sense the sequence context to regulate unwinding (dashed circle). (**b**) D10 can unwind Holliday junction-like substrates that could be established from a stalled replication fork-like substrate as in (**a**).

*In vitro*, unwinding of Y-junctions by D10 occurs with a unique polarity that appears to be established by protein-DNA base contacts (Figs 5 and 6). Furthermore, the preferential displacement of one oligonucleotide (“oligo A”) from substrate Y20 resembles the conversion of a three-way stalled replication fork to a Holliday junction via a “chicken foot” intermediate by RecG^38^,^39^. As such, the arms of this fork (Y20) can be assigned as either ‘template’, ‘leading’ or ‘lagging’ (Fig. 8). All considered, the data for D10 suggest an operating mechanism similar to RecG, proposed from the atomic structure of the *Thermotoga maritima* protein in complex with a three way DNA junction^40^: The template strand would engage with the motor or translocase module of the complex, and this fits with the observation that DNA strand discontinuity (“nicking”) of either template DNA strand at the junction of Y20 inhibits D10-dependent unwinding (Fig. 4). A DNA strand splitting “wedge” may operate at the fork junction to displace the nascent leading and lagging strand ssDNA, while all dsDNA arms of the fork are in close proximity to protein. We speculate that in D10, the wedge and sequence-sensing residues are likely to be in close proximity. The model of RecG action, and by extension D10, predicts that the enzyme can translocate on dsDNA. Although in the RecG-DNA structure the dsDNA did not extend into the ATP*ase* domain to reveal the motor in operation, the stimulation of ATP*ase* in this type of SF2 helicases by ssDNA is less marked than by dsDNA^41^,^42^, and absent in D10 (Fig. 1).

Evaluation of the available structural and biochemical data for SF2 helicases indicate that extensive phosphodiester backbone contacts are necessary for the continuity of the unwinding process while observable nucleobase contacts are not. For example, in the RecG structure aromatic side chains stabilise flipped out bases by simple planar stacking with no indication of base specificity^40^. Similarly, in the RuvA-Holliday junction structure protein-DNA contacts are restricted to the minor groove^43^. Despite this, pausing, periodic and stepping behaviour that have been observed in single molecule experiments for representative helicases from several superfamilies, not otherwise apparent in ensemble experiments, have been related to sequence context^44^,^45^,^46^. In each case though, these effects were attributed to the thermodynamic stability of the duplex nucleic acid, rather than direct enzyme-nucleic acid interactions. To date only the vaccinia virus NPH-II helicase involved in aspects of RNA metabolism has been shown to have a distinct bias for a purine-rich tracking strand although the nature of this bias remains unclear^47^.

In marked contrast to the above, our observations indicate that the DNA nucleobases influence D10 unwinding directly for the following reasons: First, minor sequence alterations at the unwinding junction result in profound changes in substrate unwinding ability while DNA binding and DNA dependent ATP*ase* are relatively unaffected, while there is no correlation between GC content and the efficiency with which a strand is displaced from a test substrate. Second, as discussed previously, the combination of OH· protection, DEPC interference and the effects of DNA strand nicking reveal a stereo-specific mode of DNA interaction that relates directly to substrate unwinding specificity. The determinant of this specificity must reside in the DNA base sequence. Protein-nucleobase contacts that determine specific protein-DNA interactions are usually confined to the DNA’s major groove^48^ and the DEPC interference assay would indicate close contacts between D10 and purine N7 atoms here.

Although our observations with D10 are based on a strand displacement assay, it is perhaps more likely that direct DNA sequence sensing by D10 (in *cis*) can terminate or pause an already productive unwinding cycle to regulate D10 branch migration. The processing of recombination intermediates by RuvABC and RecBCD is also regulated by specific sequences. In contrast to D10 however, the unwinding action of the RuvB and RecBD motors is regulated, in *trans*, by an auxiliary protein’s base-specific recognition of a DNA sequence. The RuvC endonuclease recognises and cleaves Holliday junctions when resolution hotspots are encountered^49^,^50^, while RecC interaction with the Chi (χ) sequence resets the operation of the RecBD helicase/endonuclease^51^,^52^. Similarly, the *Bacillus subtilis* AddAB helicase/nuclease, which like RecBCD is involved in DNA break processing, is regulated by the AddB subunit. In this case AddB is responsible for Chi recognition and is structurally related to the AddA SF1 helicase but catalytically inactive. Interestingly, the ssDNA-binding groove in AddB is highly modified to provide the specificity for Chi binding through base interactions^53^. Thus, in the SF2 helicase D10 it is plausible that the helicase’s ssDNA binding groove can provide base sensing without loss of the motor function.

The replication apparatus of bacteriophage T5 has not been widely studied despite its prodigious replicative capacity^22^. The previously reported T5 D2 helicase has the unusual properties of possessing a bi-polar unwinding activity and the ability of non-hydrolysable adenine nucleotides to support limited 3′–5′ helicase action^25^. Although the roles of D10 and D2 in T5 replication are unclear, we can speculate that for D10 at least, the sequence and functional similarities with UvsW contribute to the replication efficiency of T5 through RDR.

Genes capable of encoding D10 homologs are present in the genomes of viruses from eukaryotic and prokaryotic hosts as well as in archaebacteria such as the *A. fulgidus* Xpb helicase which is involved in transcription and nucleotide excision repair^54^. Recently, specific residues of the DNA binding channel of the related XPD helicase have been shown to sense damaged compared to undamaged DNA during excision repair^55^. Thus, the ssDNA-binding groove of SF1 and related SF2 helicases may have evolved a more dynamic range of DNA interacting function over non-specific phosphate backbone contacts, providing base and modified base sensing functions to regulate substrate interactions and processing during catalysis. Furthermore, our studies suggest that DNA sequence-sensing helicases with roles in DNA transcription, replication and recombination are widespread in nature and not solely confined to bacteriophages.

## Methods

### D10 Constructs

The T5 D10 gene (accession no. YP_006952) was amplified from T5 phage genomic DNA by PCR with *Pfu* DNA polymerase (Promega) using the sense primer (5′-AATTGAATTCTTAAGGTTGTTATATCTAATAAAG-3′) and antisense primer (5′-ATTTAAGCTTTTATGAGCTGTTGCCAAATGCA-3′), which included EcoRI and HindIII recognition sites, respectively (underlined). The T5 D10 mutant encoding the R389N substitution was generated by overlapping PCR using these primers together with two additional internal primers (5′-AATGTTCAACGTATTGTC-3′ and 5′-AATACGTTGAACATTGCCTGCAAGCTGTTC-3′). The position of the mutated bases are underlined. Each open reading frame (ORF) was cloned into the vector pGEX-KG^56^ and sequence integrity was confirmed using a BigDye Terminator v3.1 Cycle Sequencing Kit and ABI PRISM™ 3730 DNA Analyzer (Applied Biosystems), at the Core Genomics Facility, University of Sheffield Medical School.

### Expression and Purification of D10 Protein

Wild-type and R389N D10 glutathione-S-transferase (GST) fusion proteins were expressed in *E. coli* XL1-Blue cells cultured in 2YT media^57^ containing 100 µg/mL ampicillin at 25 °C for 8 hr following IPTG induction 0.5 mM) at A_600nm_ of 1. Cell pellets were recovered by centrifugation (10,000 x *g*, 30 min. at 4 °C) and stored at −80 °C and all the purification steps were performed at 4 °C. Cell pellets were thawed and re-suspended in 3 ml of lysis buffer (25 mM Tris-HCl pH 7.5, 1 M NaCl, 1 mM EDTA, 5 mM DTT, 10% v/v glycerol) per gramme of cells plus 1 mM phenylmethylsulfonylfluoride (PMSF), sonicated and centrifugated at 40,000 x *g* for 30 min. A 5% w/v solution of polyethylenimine (pH 8, Sigma-Aldrich, UK), was added to the supernatant to a final concentration of 0.65% w/v and the solution cleared at 25,000 x *g* for 5 min. GST-D10 protein was precipitated by addition of ammonium sulphate to 40% saturation and centrifugation at 25,000 x *g* for 30 min. The precipitate was dissolved in lysis buffer (1 ml per 10 grams of *E. coli* cells) and incubated with glutathione Sepharose beads (GE Healthcare; 1 ml of beads per 20 grams of cells) for ~18 h. Beads were washed sequentially with 50 bead volumes of lysis buffer, and 50 bead volumes of lysis buffer with 0.3 M NaCl. The GST-D10 protein was eluted with GST elution buffer (25 mM Tris-HCl pH 7.5, 0.3 M NaCl, 1 mM EDTA, 5 mM DTT, 20 mM reduced glutathione, 10% v/v glycerol) and digested with thrombin (~1 unit per g of *E. coli* cells) for ~18 h to cleave off the GST fusion partner. The protein was further purified by cation exchange chromatography (Source S (GE Healthcare)), 10 mM sodium phosphate pH 6.5, 1 mM EDTA, 5 mM DTT, 10% v/v glycerol, 0.2-0.7 M NaCl gradient) and gel filtration (Superdex 75 (GE Healthcare), 20 mM Tris-HCl pH 7.5, 0.3 M NaCl, 1 mM EDTA, 1 mM PMSF, 5 mM DTT, 10% v/v glycerol). Peak fractions were pooled, concentrated, dispensed into aliquots and stored at −80 °C. D10 protein concentration was determined in the presence of 7 M guanidine hydrochloride by UV spectrophotometry using a molar extinction coefficient of 58330 M^−1^ cm^−1^.

### ATP*ase* Assays

ATP*ase* assays were performed in 20 mM HEPES-NaOH pH 7.5, 120 mM NaCl, 0.1% v/v NP40 alternative (Calbiochem, UK), 0.1 mg/ml BSA, 2 mM DTT, 0.0125 μM [γ-^32^P]ATP (6000 Ci/mmol), 5 mM MgCl_2_, 5 mM ATP at 22 °C for 10 min, unless stated otherwise. The release of radioactive phosphate was measured using the charcoal-binding assay of Iggo and Lane^58^. All data are from a minimum of three repeats and the mean is shown with the standard deviation (SD) delimited by the error bars.

### Helicase and electrophoretic mobility shift assays (EMSA)

The DNA substrates were constructed by annealing the synthetic oligonucleotides (Table S1 and Figure S7). The oligonucleotides were labeled at the 5′ end with T4 polynucleotide kinase and [γ-^32^P]ATP (6000 Ci/mmol) as indicated in each figure using the protocol previously described^34^. The labelled substrates were resolved and purified from 8% (19:1) polyacrylamide gels and quantified based on the specific activity of the component oligonucleotides^34^.

Helicase assays (0.1 nM substrates) were performed in 20 mM HEPES-NaOH pH 7.5, 20 mM NaCl, 0.1% v/v NP40 alternative, 0.1 mg/ml BSA, 2 mM DTT, 5 mM MgCl_2_, 5 mM ATP at 37 °C for 20 min and terminated with 6 x stop buffer (120 mM EDTA, 0.6% w/v SDS, 1% w/v bromophenol blue, 60% v/v glycerol). Products were separated on 8% (19:1) polyacrylamide gels containing 0.05% w/v SDS, using 1 x TBE/0.05% w/v SDS running buffer, visualized and quantified by phosphorimaging. Strand annealing assays were performed under the same reaction conditions with or without the addition of ATP as indicated and processed in the same way as helicase assays.

DNA binding reactions (0.1 nM substrates) were performed in 20 mM HEPES-NaOH pH 7.5, 135 mM NaCl, 0.1% v/v NP40 alternative, 0.1 mg/ml BSA, 2 mM DTT, 1 mM EDTA, 10% v/v glycerol at 22 °C for 20 min. The binding reactions were resolved on 6% (30:1) polyacrylamide gels in 0.25 x TBE buffer, visualized and quantified as above. Competition assays with poly d(AT) competitor DNA were performed under the same reaction conditions with radiolabeled substrate and competitor DNA mixed before the addition of protein (concentrations indicated in the figure legends). All graphed data shown are from a minimum of three repeats. Data points show the mean and the standard deviation (SD) delimited by the error bars.

### Hydroxyl Radical Footprinting

Hydroxyl radical footprinting in solution was carried out essentially as described previously^59^. The binding reactions (50 μl, containing 10 nM of Y20 substrate with one strand ^32^P end-labelled) were set up as described for the EMSA, except that glycerol was omitted. Following a 20 minute incubation at 22 °C, the hydroxyl radical was generated by addition of 0.375 mM sodium ascorbate, 0.0275% w/v H_2_O_2_, 1 mM (NH_4_)_2_FeSO_4_·6H_2_O and 2 mM EDTA for 2 min and quenched with 0.25 volume of 200 mM thiourea. Cleaved products were extracted twice with phenol/chloroform and analyzed on 15% (19:1) polyacrylamide gels containing 8 M urea in 1 x TBE buffer, after adding an equal volume of 98% formamide loading buffer and heating.

### DEPC Interference

The Y20 substrates for DEPC interference were prepared by 5′ end-labelling one strand with ^32^P and modifying it with diethylpyrocarbonate (DEPC) essentially as described^30^,^60^. Approx. 60 ng of end-labelled DNA was carbethoxylated by adding 4 μl of DEPC in 200 μl of cacodylate buffer (50 mM sodium cacodylate pH 7.0, 1 mM EDTA). After incubation at 37 °C for 20 min, the DNA was ethanol precipitated twice and annealed with its complementary oligonucleotides, before gel-purification and quantification as described above. Helicase reactions contained 5 nM substrate and 0.005 nM D10. To generate DEPC interference data for the unwinding reaction, helicase assays were set up so that only a small fraction of the substrate (~17%) was unwound. After electrophoresis, the reaction products were detected by autoradiography and the bands excised from the gel. The DNA was soak-eluted in 1 x TAE buffer (40 mM Tris acetate, 1 mM EDTA) at 4 °C overnight and recovered by phenol/chloroform extraction, ethanol precipitation, and cetyltrimethylammonium bromide precipitation. The DNA was then cleaved with piperidine, extracted with butanol and ethanol precipitated. Finally, the products were analyzed on urea-polyacrylamide sequencing gels, as described above.

## Additional Information

**How to cite this article**: Wong, I. N. *et al*. Bacteriophage T5 gene D10 encodes a branch-migration protein. *Sci. Rep.* **6**, 39414; doi: 10.1038/srep39414 (2016).

**Publisher's note:** Springer Nature remains neutral with regard to jurisdictional claims in published maps and institutional affiliations.

## Supplementary Material

Supplementary Movie

Supplementary Information
