# Supplementary material for: Bacteriophage T5 gene D10 encodes a branch-migration protein
Source: Sci Rep. 2016 Dec 23;6:39414. doi: 10.1038/srep39414 (PMC5180179; doi:10.1038/srep39414)
Supplement: Supplementary Information [file srep39414-s2.pdf]

Supplementary Information for:

Bacteriophage T5 gene D10 encodes a branch-migration protein

Authors: Io Nam Wong, Jon R. Sayers and Cyril M. Sanders

Supplementary Table S1

Supplementary Figure S1

Supplementary Figure S2

Supplementary Figure S3

Supplementary Figure S4

Supplementary Figure S5

Supplementary Figure S6

Supplementary References

ID	Sequence (5’-3’)
Oligo A	TTCGAAACGTGCGCGCCCATACGCCAAGCTTAACGGTGAT
Oligo A’	TTCGAAACGTGCGCGCCTCGATGCCAAGCTTAACGGTGAT
Oligo A’’	TTCGAAACGTGCGGAATCATACGGGCGGCTTAACGGTGAT
Oligo a1	TTCGAAACGTGCGCGCCCAT
Oligo a2	ACGCCAAGCTTAACGGTGAT
Oligo B	ATCACCGTTAAGCTTGGCGTCGAATTCGAGCTCGGTACCC
Oligo B’	ATCACCGTTAAGCTTGGCATACGATTCGAGCTCGGTACCC
Oligo B’’	ATCACCGTTAAGCCGCCCGTCGACCAAGAGCTCGGTACCC
Oligo b1	ATCACCGTTAAGCTTGGCGT
Oligo b2	CGAATTCGAGCTCGGTACCC
Oligo C	GGGTACCGAGCTCGAATTCGATGGGCGCGCACGTTTCGAA
Oligo C’	GGGTACCGAGCTCGAATCGTCGAGGCGCGCACGTTTCGAA
Oligo C’’	GGGTACCGAGCTCTTGGTCGATGATTCCGCACGTTTCGAA
Oligo c1	GGGTACCGAGCTCGAATTCG
Oligo c2	ATGGGCGCGCACGTTTCGAA
Oligo D	TTCGAAACGTGCGCGCCCATACGCCAAGCTTAACGGTGATTTTGGTTTTCCCGCTTGAAAAACCG GCAACGGTGT
Oligo E	ACACCGTTGCCGGTTTTTCAAGCGGGAAAACCAAAATCACCGTTAAGCTTGGCGTCGAATTCGAG CTCGGTACCC
Oligo F	GGGTACCGAGCTCGAATTCGTGGGCGGTGCCCAACGCATA
Oligo G	TATGCGTTGGGCACCGCCCAATGGGCGCGCACGTTTCGAA
Oligo H	GGGTACCGAGCTCGAATTCGTGGGCGGTGCCCAACGCATATATTACTGTCGGAGTATCAAACAG TTTTCGCGTCA
Oligo I	TGACGCGAAAACTGTTTGATACTCCGACAGTAATATATGCGTTGGGCACCGCCCAATGGGCGCG CACGTTTCGAA
Oligo J	TTCGAAACGTGCGCGCCCATATCGTTACATTAGAAGGATCCACTGGTTTCCCGCTTGAAAAACCG GCAACGGTGT
Oligo K	ACACCGTTGCCGGTTTTTCAAGCGGGAAACCAGTGGATCCTTCTAATGTAACGATCGAATTCGAG CTCGGTACCC
Oligo L	GGGTACCGAGCTCGAATTCGATCGTTACATTAGAAGGATCCACTGGTTTCCCGCTTGAAAAACCG GCAACGGTGT
Oligo M	ACACCGTTGCCGGTTTTTCAAGCGGGAAACCAGTGGATCCTTCTAATGTAACGATATGGGCGCGC ACGTTTCGAA
Oligo N	$(T)_{20}$
Oligo O	$(T)_{55}$
Oligo P	$(T)_{20}$CGAATTCGAGCTCGGTACCC
Oligo Q	GGGTACCGAGCTCGAATTCG$(T)_{20}$
Oligo R	$(T)_{55}$CGAATTCGAGCTCGGTACCC
Oligo S	GGGTACCGAGCTCGAATTCG$(T)_{55}$
Oligo T	GGGTACCGAGCTCGAATTCGTTTTTTTTTTTTTTTTTTTTATGGGCGCGCACGTTTCGAA
Oligo U	TTCGAAACGTGCGCGCCCATAAAAAAAAAAAAAAAAAAAACGAATTCGAGCTCGGTACCC

Table S1. Oligonucleotides used in this study.

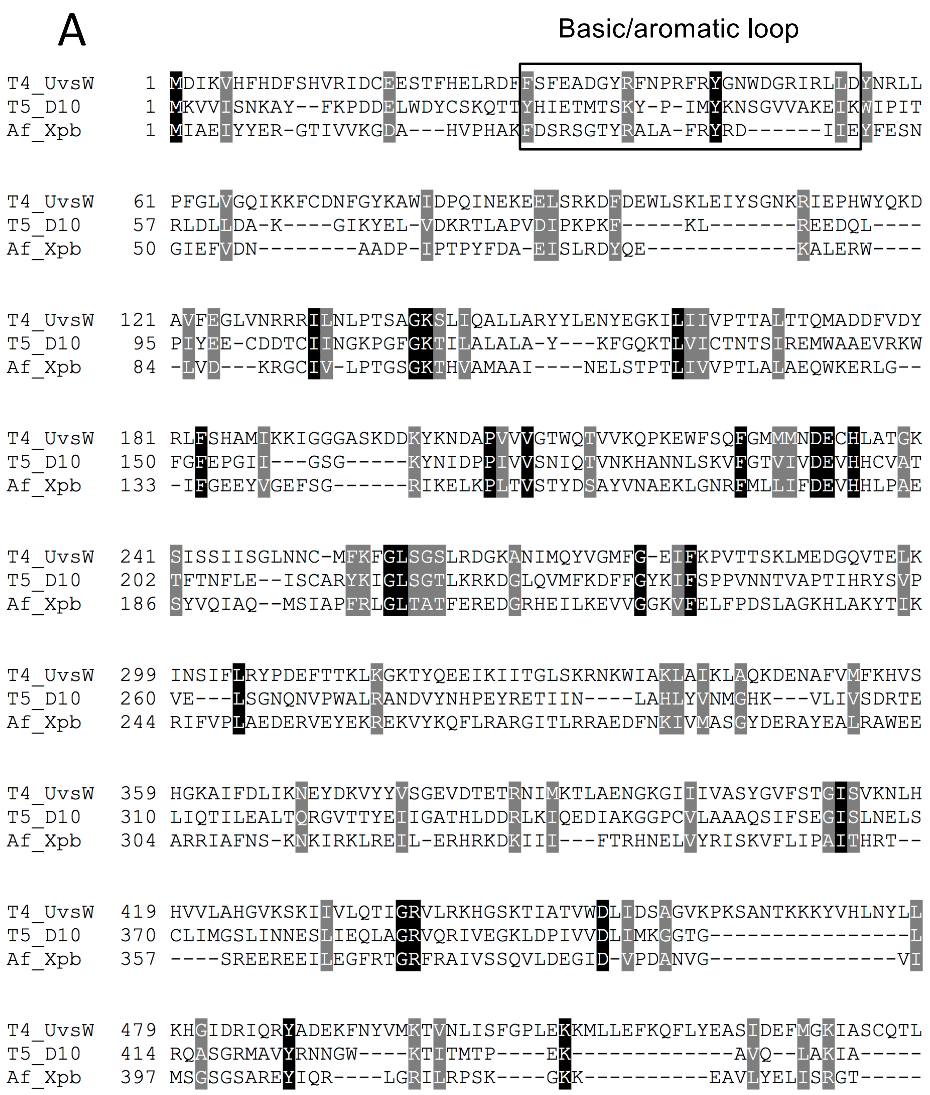

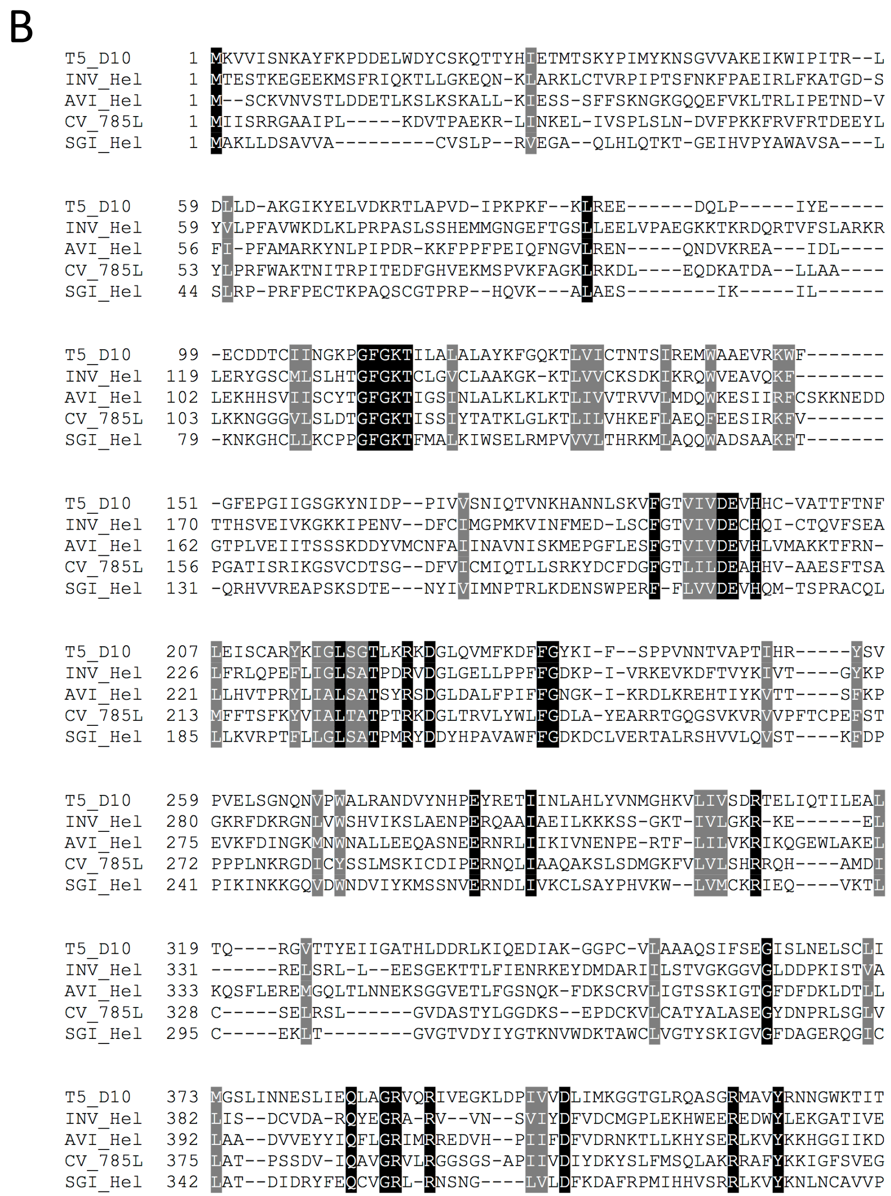

Supplementary Figure S1 Sequence alignments showing D10 homologs. D10 homologs identified by BLAST search (Altschul, S. F., Gish, W., Miller, W., Myers, E. W., & Lipman, D. J. (1990). Basic local alignment search tool. J Mol Biol, 215(3), 403-410. doi: 10.1016/S0022-2836(05)80360-2) were aligned using Multalin (Corpet, F. (1988). Multiple sequence alignment with hierarchical clustering. Nucleic Acids Res, 16(22), 10881-10890) and the alignments rendered using Boxshade (http://www.ch.embnet.org/software/BOX_form.html). **(A)** UvsW from bacteriophage T4 (P11107) and *Archaeoglobus fulgidus* XpB protein (WP_048064221). Identical residues shown in black boxes, similar in grey. **(B)** Representative alignment of D10 protein (T5_D10) with gene products encoded by viruses infecting eukaryotic organisms; CV_785L, *Acanthocystis turfacea* Chlorella virus 1 (AGE56747); INV_Hel, *Eristalis tenax* Insectomime virus (AHA46029); AVI_HEL, *Armadillidium vulgare* iridescent virus (YP_009046669); and SGI_Hel, Singapore grouper iridovirus (YP_164247).

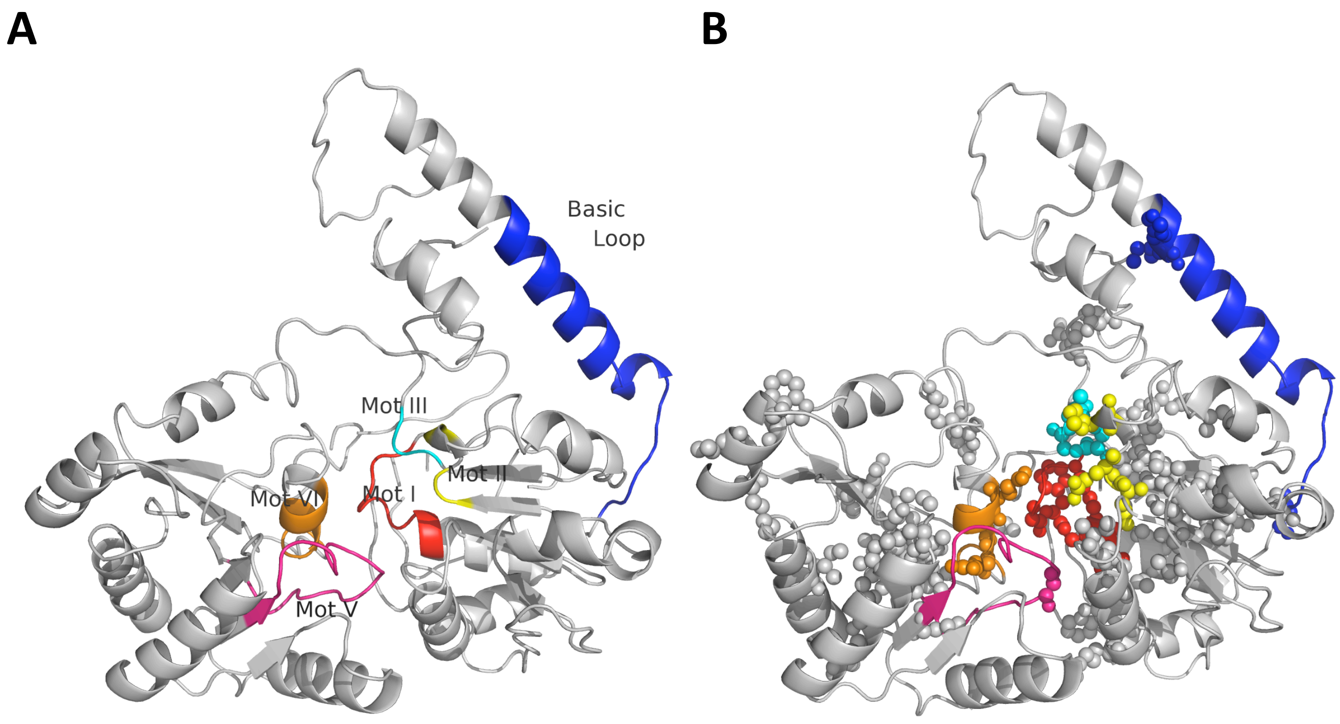

Supplementary Figure S2 Predicted structure of D10 protein. (**A**) SF2 motifs coloured red (I), yellow (II), cyan (III), magenta (V) and orange (VI). The conserved basic/aromatic loop is shown in blue. (**B**) residues conserved between T5 D10 and homologues identified in eukaryotic viruses are shown as spheres. See also the Supplementary Movie file. Structure modelled using Phyre2 (The Phyre2 web portal for protein modeling, prediction and analysis. 2015. Kelley, L.A., Mezulis, S., Yates, Y.M., Wass, M.N., Sternberg, M.J.E., Nature Protocols 10, 845-858).

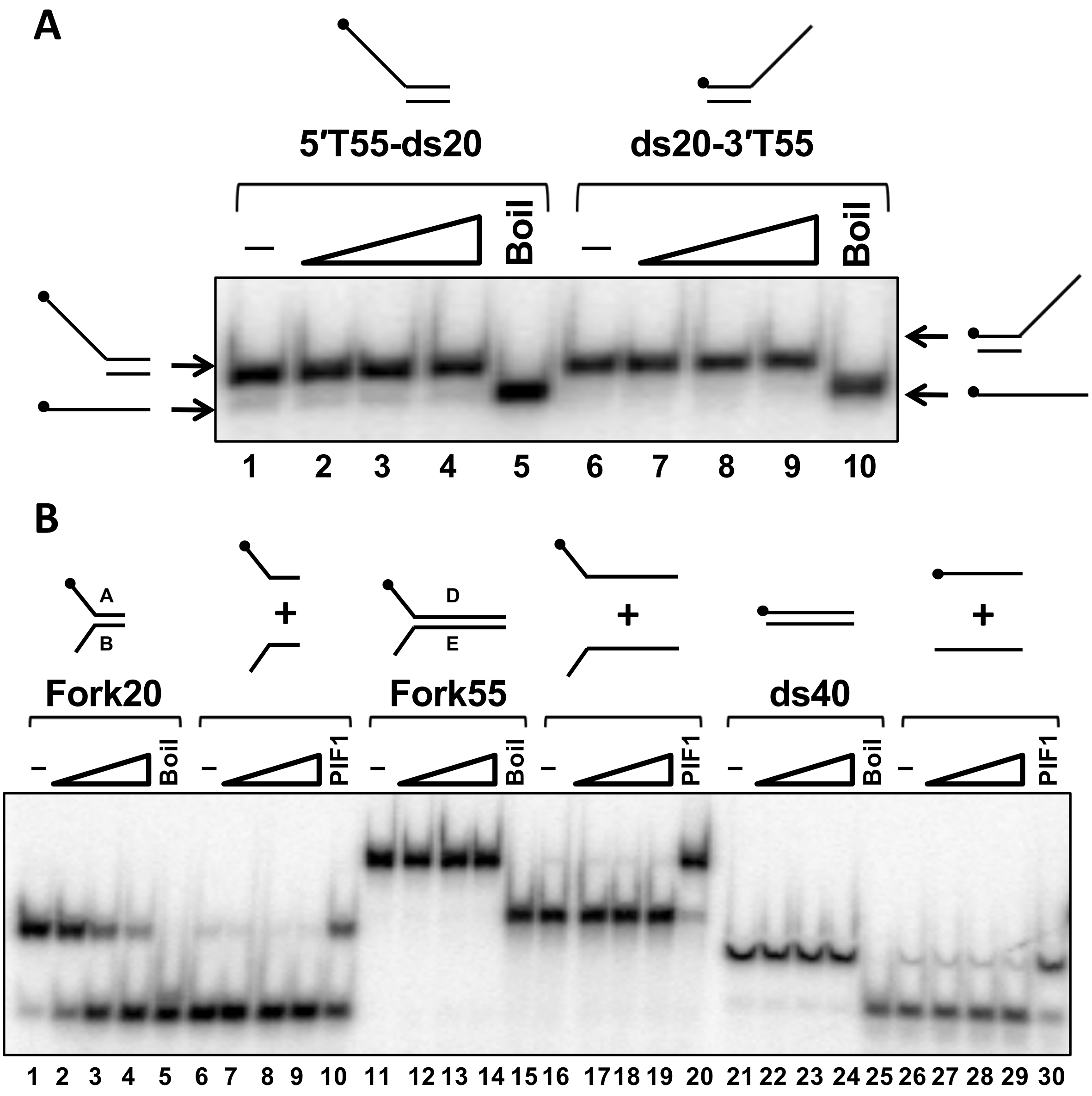

Supplementary Figure S3. Unwinding and strand annealing activity of D10 using linear double and partially single- and double-stranded DNA substrates. (**A**) Helicase assays with 5′T55-ds20 and ds20-3′T55 (0.1 nM) and D10 (0.01, 0.04 and 0.16 nM) were performed as described in experimental procedures. Lanes 1 and 6, no protein control (-); Lanes 5 and 10, heat-denatured substrate control (Boil); Lanes 2-4 and Lanes 7-9, increasing D10 protein concentration. These simple partially single- and double-stranded substrates were not unwound by D10 even at enzyme concentrations up to 100 nM (not shown) under the same reaction conditions. (**B**) Strand annealing activity was undetectable (0.1 nM substrates and 0.01, 0.04 and 0.16 nM, same reaction conditions as helicase assays). Lanes 1-5, unwinding of substrate Fork20. In lanes 6-10, the Fork20 substrate was heat denatured before addition to the reactions; no D10 catalysed strand annealing was observed (lanes 7-9), but strand annealing was catalysed by the human PIF1 helicase[34], lane 10. Lanes 11-20 and Lanes 21-30 as 1-10 except using substrates Fork55 with the longer complementary arms and ds40 without unpaired tails, respectively.

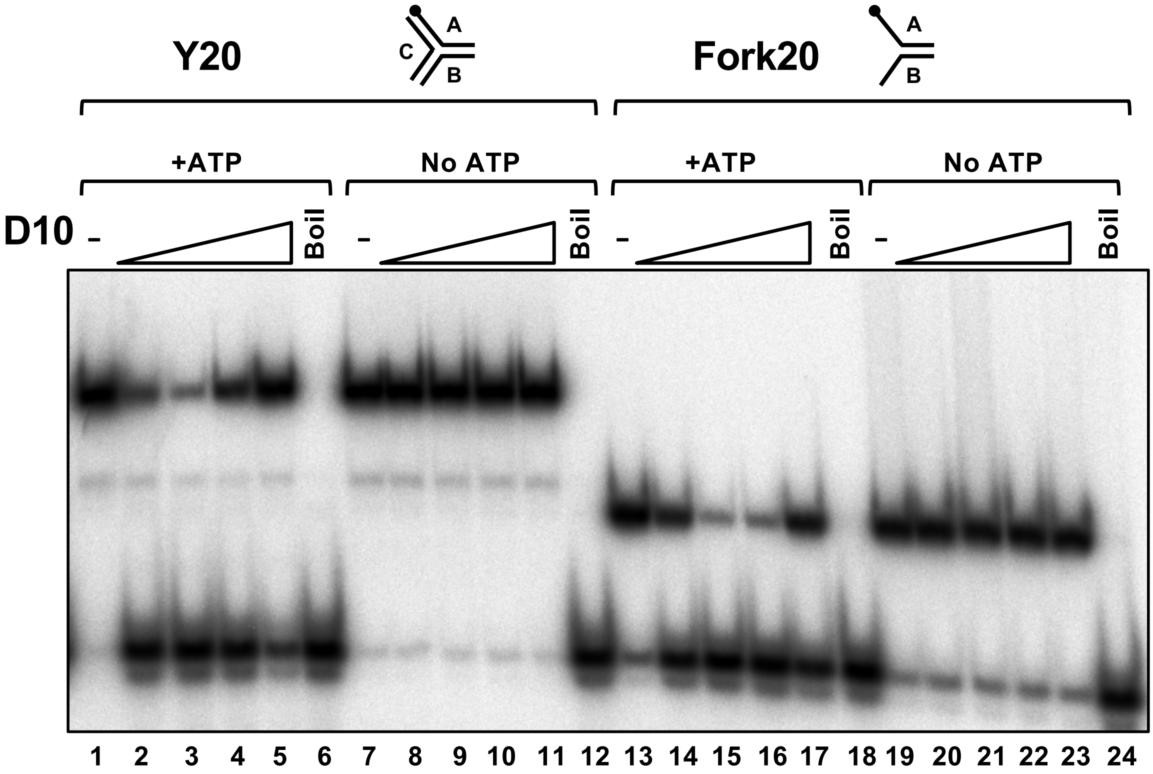

Supplementary Figure S4 ATP is required for unwinding single- and double-stranded fork-like DNA substrates. (**A**) Helicase assays with substrates Y20 and Fork20 (0.1 nM) and D10 (0.01, 0.04 and 0.16 nM and 0.32 nM) were performed as described in experimental procedures, with and without ATP. No significant unwinding activity was observed for either substrate in the absence of ATP in three independent experiments. Lanes 1, 7, 13 and 19, no protein control (-); Lanes 6, 12, 18 and 24, heat-denatured substrate control (Boil); Lanes 2-5, 8-11, 14-17 and 20-23, increasing D10 protein concentration.

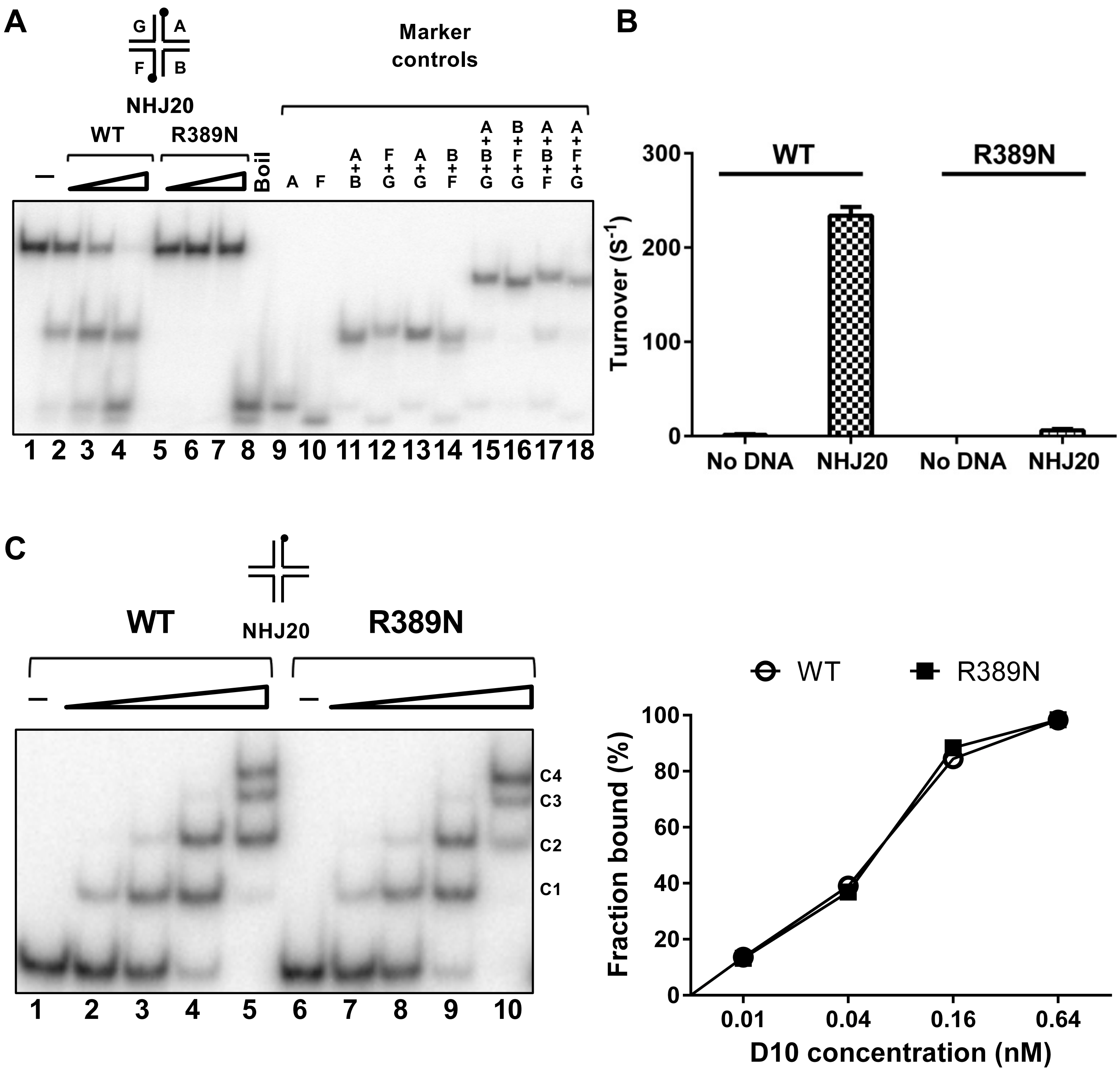

Supplementary Figure S5. Helicase, ATP*ase* and DNA-binding activities of D10 R389N using the non-homologous Holliday junction substrate NHJ20. Helicase activity (0.1 nM NHJ20, 0.01, 0.04 and 0.16 nM D10) (**A**) and ATP*ase* activity (**B**) were almost completely abolished by the arginine finger R389N substitution. In **A,** lanes 9-18 are labelled DNA markers for all possible products. Lane 1, no protein control; lane 8, heat-denatured substrate (Boil). (**C**) D10 R389N has similar DNA binding activity to that of wild-type D10 (0.1 nM NHJ20, 0.01, 0.04, 0.16 and 0.64 nM D10 or mutant).

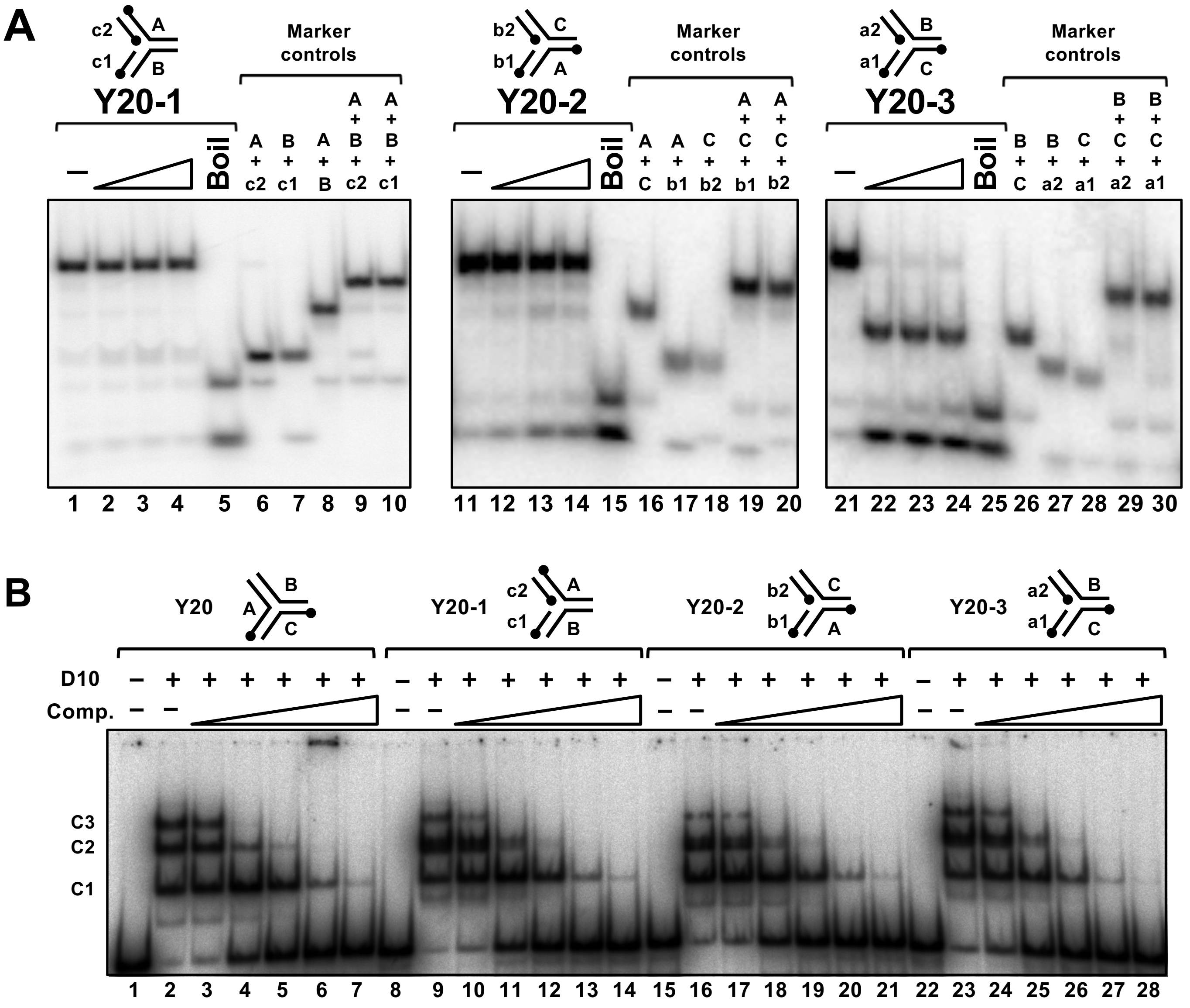

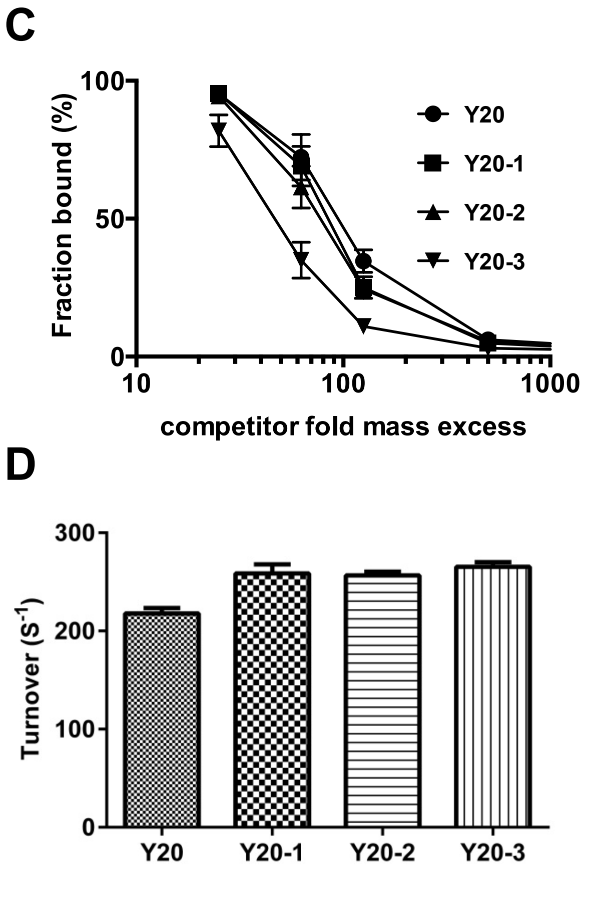

Supplementary Figure S6 Helicase, DNA-binding and ATP*ase* activities of D10 using Y20, with and without a nicked oligonucleotide. (**A**) Unwinding of the nicked Y20 substrates Y20-1 (oligo C nicked), Y20-2 (oligo B nicked) and Y20-3 (oligo A nicked) by D10. Lanes 1, 11 and 21, no protein control (-); lanes 5, 15 and 25, heat-denatured substrate control (Boil); lanes 2-4, lanes 12-14 and lanes 22-24, with D10 (0.01, 0.04 and 0.16 nM); lanes 6-10, 16-20 and 26-30, markers for possible products. (**B**) Competition binding assays with Y-junction substrates (0.1 nM substrate, 0.4 nM D10, 2-200ng poly d(AT) competitor) were performed as described in Figure 7, main manuscript. (**C**) Quantification of the data in (B), n=4, mean and standard deviation. Only binding to the substrate Y20-3, equivalent to a nick in olgo A, showed an increased sensitivity to the addition of competitor (~2 fold) indicating a small reduction in D10 binding affinity. (**D**) D10 ATP*ase* activity stimulated by the four Y-junctions substrates. Reactions (37°C for 20 min) were performed with 20 mM NaCl in the buffer.

Linear DNA substrates

ssDNA

T20 — Oligo N: 5′ TTTTTTTTTTTTTTTTTT 3′

T55 — Oligo O: 5′ TTTTTTTTTTTTTTTTTTTTTTTTTTTTTTTTTTTTTTTTTTTTTT 3′

dsDNA

ds20 — Oligo b2 / Oligo c1:
```
5′ CGAATTCGAGCTCGGTACCC 3′
3′ GCTTAAGCTCGAGCCATGGG 5′
```

ds60 — Oligo T / Oligo U:
```
5′ GGGTACCGAGCTCGAATTCGTTTTTTTTTTTTTTTTTTTTTATGGGCGCGCACGTTTCGAA 3′
3′ CCCATGGCTCGAGCTTAAGCAAAAAAAAAAAAAAAAAAAAATACCCGCGCGTGCAAAGCTT 5′
```

5psT20/5pST55 — Oligo P/R / Oligo c1:
```
5′ (T)20/55 CGAATTCGAGCTCGGTACCC 3′
            3′ GCTTAAGCTCGAGCCATGGG 5′
```

3psT20/3psT55 — Oligo Q/S / Oligo b2:
```
5′GGGTACCGAGCTCGAATTCG(T)20/55  3′
3′CCCATGGCTCGAGCTTAAGC 5′
```

Fork DNA substrates

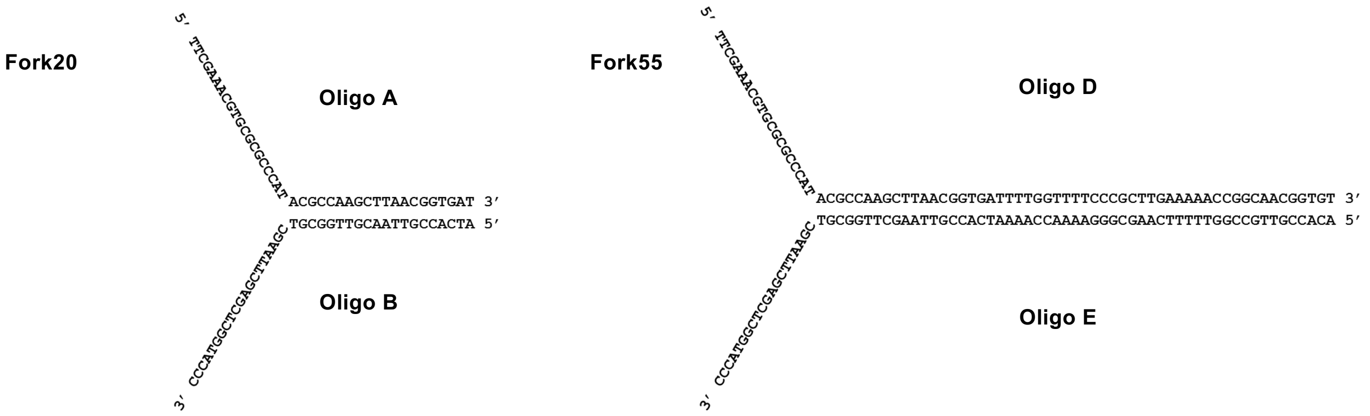

Y-junction substrates

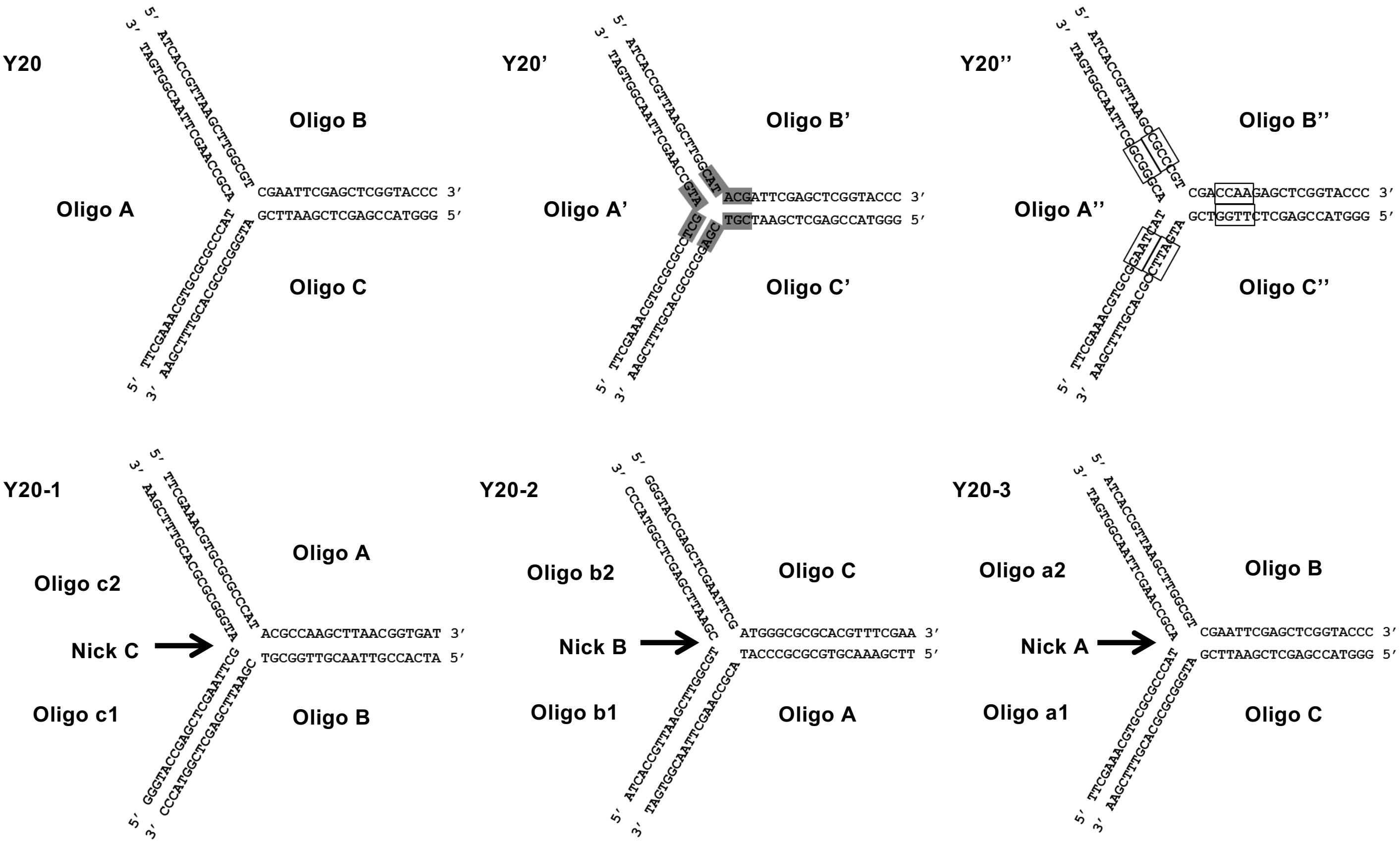

Supplementary Figure S7. Oligonucleotide substrates used in this study.

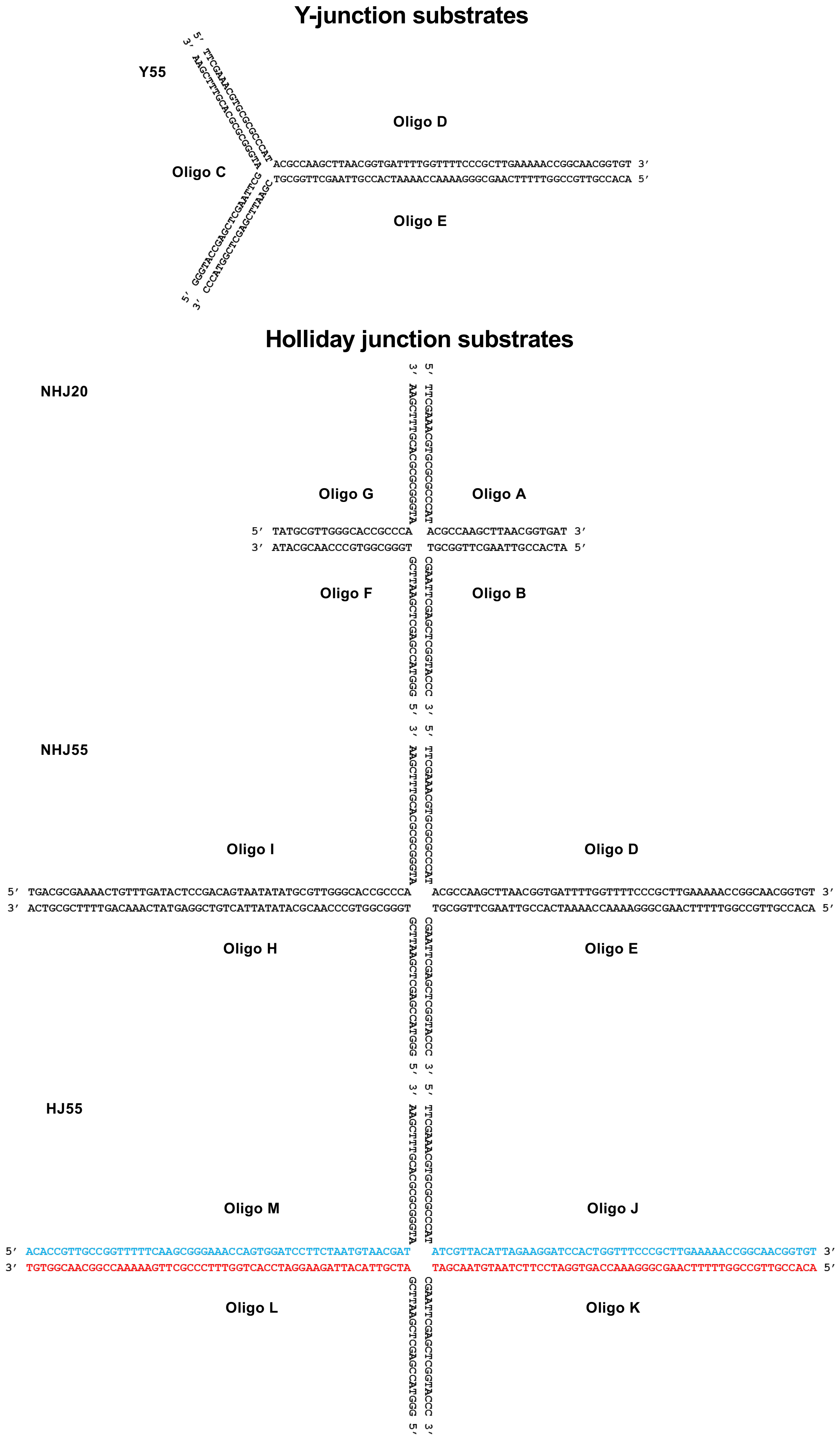

Supplementary Figure S6. Oligonucleotide substrates used in this study (continued).

References

1. Altschul, S.F., Gish, W., Miller, W., Myers, E.W. and Lipman, D.J. (1990) Basic local alignment search tool. *J. Mol. Biol.*, **215**, 403-410.

2. Corpet, F. (1988) Multiple sequence alignment with hierarchical clustering. *Nucleic Acids Res*, **16**, 10881-10890.

3. Kelley, L.A. and Sternberg, M.J. (2009) Protein structure prediction on the Web: a case study using the Phyre server. *Nat Protoc*, **4**, 363-371.
